# Evaluating a learning health system initiative: Lessons learned during COVID‐19 in Saskatchewan, Canada

**DOI:** 10.1002/lrh2.10350

**Published:** 2022-10-09

**Authors:** Gary Groot, Stephanie Witham, Andreea Badea, Susan Baer, Michelle Dalidowicz, Bruce Reeder, John Froh, Tracey Carr

**Affiliations:** ^1^ Department of Community Health and Epidemiology University of Saskatchewan Saskatoon Saskatchewan Canada; ^2^ Saskatchewan Health Authority Royal University Hospital Saskatoon Saskatchewan Canada; ^3^ Saskatchewan Health Authority Health Sciences Library Regina Saskatchewan Canada

**Keywords:** COVID‐19, decision‐making, evaluation, health services research, learning health system, Saskatchewan

## Abstract

**Introduction:**

Evaluating a learning health system (LHS) encourages continuous system improvement and collaboration within the healthcare system. Although LHS is a widely accepted concept, there is little knowledge about evaluating an LHS. To explore the outputs and outcomes of an LHS model, we evaluated the COVID‐19 Evidence Support Team (CEST) in Saskatchewan, Canada, an initiative to rapidly review scientific evidence about COVID‐19 for decision‐making. By evaluating this program during its formation, we explored how and to what extent the CEST initiative was used by stakeholders. An additional study aim was to understand how CEST could be applied as a functional LHS and the value of similar knowledge‐to‐action cycles.

**Methods:**

Using a formative evaluation design, we conducted qualitative interviews with key informants (KIs) who were involved with COVID‐19 response strategies in Saskatchewan. Transcripts were analyzed using reflexive thematic analysis to identify key themes. A program logic model was created to represent the inputs, activities, outputs, and outcomes of the CEST initiative.

**Results:**

Interview data from 11 KIs were collated under three overarching categories: (1) outputs, (2) short‐term outcomes, and (3) long‐term outcomes from the CEST initiative. Overall, participants found the CEST initiative improved speed and access to reliable information, supported and influenced decision‐making and public health strategies, leveraged partnerships, increased confidence and reassurance, and challenged misinformation. Themes relating to the long‐term outcomes of the initiative included improving coordination, awareness, and using good judgment and planning to integrate CEST sustainably into the health system.

**Conclusion:**

This formative evaluation demonstrated that CEST was a valued program and a promising LHS model for Saskatchewan. The future direction involves addressing program recommendations to implement this model as a functional LHS in Saskatchewan.

## 1 INTRODUCTION

Systematically analyzing and translating health system data in the dynamic knowledge generation and exchange ecosystem of a learning health system (LHS)^1^, ^2^, ^3^ can optimize health system productivity and improve patient outcomes.^4^, ^5^, ^6^, ^7^ After the Institute of Medicine first introduced the idea of an LHS in 2007 as applying available evidence to increase system effectiveness and efficiency,^2^ various conceptual frameworks have been described in the literature.^1^, ^6^, ^8^, ^9^ Since then, the LHS concept has become widely accepted and acknowledged globally.^3^, ^8^, ^9^, ^10^, ^11^ Although an LHS may address the need to translate knowledge into practice more efficiently, few studies have evaluated the process and outcomes of a “real‐life” LHS in healthcare.^1^, ^3^ Barriers to evaluating LHS programs could be several: financial constraints,^3^, ^11^ time constraints,^12^ lack of information technology,^13^ the availability and quality of data,^1^, ^3^ program commitments,^1^ and difficulty with adopting change in organizations.^11^, ^13^ However, those who have evaluated the process, outcomes, and impacts of LHS programs have demonstrated that an LHS can improve program development, patient outcomes, as well as time and cost‐effectiveness.^1^, ^6^, ^7^, ^11^, ^14^, ^15^ Evaluating an LHS encourages continuous system improvement and collaboration within the healthcare system.

Rapid assessment and dissemination of scientific evidence were needed to guide decision‐making during the emergence and ongoing severity of the COVID‐19 pandemic.^4^, ^16^ At the onset, knowledge about the virus was lacking and at times contradictory, creating uncertainty for clinicians and decision‐makers.^5^, ^17^ Early research in particular had varying degrees of scientific rigor and maturity, which quickly overwhelmed traditional scientific processes.^17^ To address this, regional, national, and international COVID‐19 initiatives were established around the world to answer pressing health‐system COVID‐19 questions.^4^, ^5^, ^6^, ^7^, ^8^, ^9^, ^10^, ^11^, ^12^, ^13^ LHS models have shown to bridge gaps in understanding, support clinical practice, improve health system efficiency, and reduce costs.^8^, ^11^, ^14^, ^18^ Scientific evidence may result in clinical practice changes during the pandemic.^5^, ^6^

One such LHS initiative to address the COVID‐19 pandemic in Saskatchewan, Canada, was the COVID‐19 Evidence Support Team (CEST), or the “Think Tank” as it is more commonly known.^19^ Academics, researchers, librarians, clinicians‐scientists, and policy‐makers from the University of Saskatchewan (USASK), Saskatchewan Health Authority (SHA), Health Quality Council (HQC), and Ministry of Health (MoH) collaborated to provide evidence for informed decision‐making.^19^ The purpose of this article is to describe a formative evaluation of the CEST initiative to assess its implementation and impact, and to explore how it might develop into a sustainable LHS for Saskatchewan.

### 1.1 Research aims

In this study, we gathered the experiences of CEST users to ask the following evaluation questions:

1. How and to what extent was the information from the CEST initiative used?
2. What were the perceptions and experiences from CEST users regarding its role as an LHS?
3. In what ways could the CEST initiative continue to be applied as a functional LHS in Saskatchewan?

## 2 METHODS

### 2.1 Program description

At the beginning of the pandemic, the first author and clinician‐scientist (GG), recognized the need for a reliable evidence‐based system to support decision‐makers, clinicians, and healthcare leaders. As a result, the CEST was established with an oversight committee consisting of members from the Emergency Operations Committee (EOC), USASK, and the Public Health Incident Command Center (PHICC). The EOC, Saskatchewan's COVID‐19 decision‐making body, used synthesized evidence from CEST to support decisions at the pandemic's onset where there was little or conflicting evidence.^19^ By coordinating weekly with the EOC chair, CEST's objectives were to (1) establish a rapid review process to support decision‐making, (2) create an online repository for knowledge sharing, and (3) initiate a learning health system in the province.^19^ Clinical experts assessed the quality and confidence of the literature, and in the case of uncertainty about a topic, CEST conducted iterative “evergreen” reviews. Three distinct CEST processes evaluated the evidence and created outputs: (1) team question prioritization, (2) question refinement with the assistance of clinical experts, library staff, and master's or PhD‐level researchers to identify a search strategy, and (3) literature synthesis for clinical experts. Of 108 COVID rapid reviews produced between March 2020 and March 2021, the typical turnaround time for the team was 21 days, with 20 completed in less than 1 day and 44 within 2 to 10 days.^19^ Reviews were sent to local MHOs and condensed findings were sent to key decision‐makers. Reviews included EOC priority questions such as: “what is the evidence for the effectiveness of universal mask use by the public?” and “what public health interventions are effective in reducing the burden of COVID‐19 disease in comparable jurisdictions to Saskatchewan?” By April 27, 2022, the COVID‐19 online repository (https://saskhealthauthority.libguides.com/covid-19/repository/home) had completed a total of 171 reviews for 11 different research teams and produced 430 documents and tables, and updated 16 “evergreen” reviews since the Spring of 2020.^20^ Additional background on the development of the initiative and its relationship to an LHS is described in a previous publication.^19^

### 2.2 Study design

A formative evaluation design was chosen to assess the experiences of Saskatchewan health system leaders who used CEST during the COVID‐19 pandemic. Used to understand and improve implementation during program development,^21^, ^22^, ^23^ formative evaluation enables the explicit study of the complexity of implementation projects and highlights answers about context, adaptations, and responses to change.^23^ This evaluation type differs from summative evaluation by identifying factors that influence the implementation of a program during its development to improve it,^24^ as opposed to evaluating the end result of a program's effectiveness.^24^ In doing so, changes can be identified before the end of a study period which can improve the long‐term effectiveness of a program.^25^

### 2.3 Data collection

We invited 13 key informants (KIs) who were involved in various public health strategies for the Saskatchewan COVID‐19 response to be interviewed for the study. Participants were selected if they were primary users of CEST and had a role in decision‐making during the pandemic. Using a semi‐structured interview guide, a researcher (SW) asked the participants about the extent to which they used CEST in their work or practice, how CEST may have affected their decision‐making or provided value to their practice, whether CEST facilitated communication between health system partners, and what future application they envisioned. Prior to the interview, participants were briefed on the study objectives and written or verbal informed consent was obtained. Interviews were conducted between September and November 2021 via WebEx or Zoom and lasted an average of 15 min (7‐22 min). Following the interview, the recordings were transcribed verbatim by the same researcher and housed in NVivo (Version 12) for subsequent analysis. The protocols for this study were reviewed and approved for an Ethics Exemption by the USASK Behavioural Research Ethics Board for the purpose of program quality improvement (TCPS Article 2.2).^26^

### 2.4 Data analysis

**FIGURE 1:**
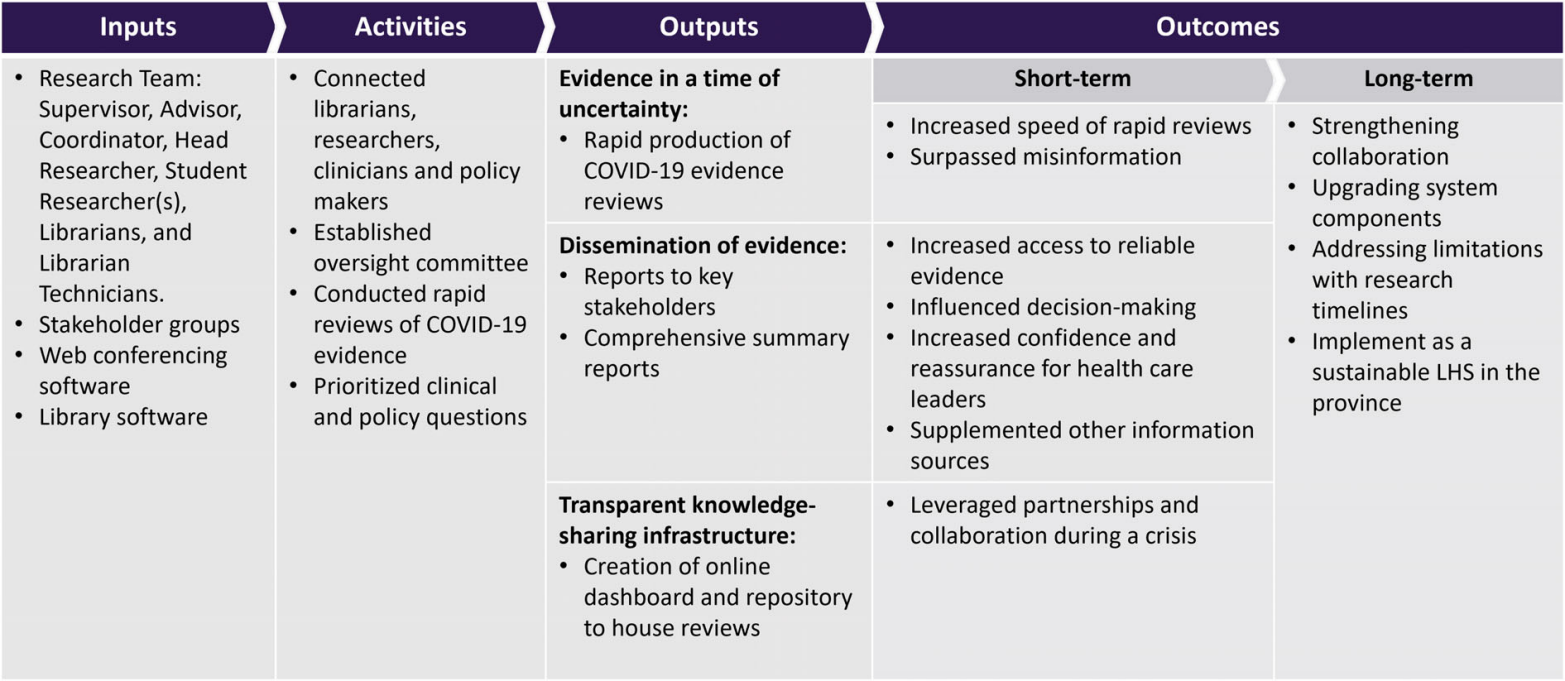
Logic model

Guided by our three research aims, we conducted a reflective thematic analysis.^27^, ^28^ Two researchers (SW and TC) followed Braun and Clarke's^27^, ^29^ six‐phase approach: (1) familiarization, (2) generating codes, (3) searching for themes, (4) revising themes, (5) defining and naming themes, and (6) producing final product or codebook.^27^ A combination of deductive and inductive coding was applied to the data to construct themes related to the impact of CEST and how it was used by the participants. Both researchers reflected on the conceptualization of codes and made revisions iteratively to develop a codebook. A program logic model (PLM) was developed to illustrate CEST's composition and its deliverables,^19^ and the program's outputs and outcomes described by KIs (Figure 1). The PLM illustrates how CEST's outputs were influenced by the activities (ie, rapid reviews) and the outcomes were associated with the outputs of the program.

## 3 RESULTS

Of the 13 participants that were contacted, 11 (84.6%) agreed to be interviewed. The informants (4 male and 7 female) included four medical health officers, three SHA directors, two physician leaders, an epidemiologist, and one chief nursing officer. Participants were from Saskatchewan's two largest cities, Regina (*n* = 5) and Saskatoon (*n* = 5), and an area in southern Saskatchewan (*n* = 1). Twelve themes were identified from the interviews: three associated with outputs, five associated with short‐term outcomes, and four associated with long‐term outcomes of the program. The long‐term outcomes are summarized using key informant recommendations for the future application of CEST.

### 3.1 Outputs

Various participants discussed their experience with the CEST deliverables and how they were used in their practice. Three main output‐related themes were identified: (1) the production of evidence in a time of uncertainty, (2) the dissemination of evidence to stakeholders, and (3) the establishment of transparent knowledge‐sharing (Table 1).

**TABLE 1:** Outputs of CEST and exemplar quotes

Section	Outputs	Exemplar quote
3.1.1	Evidence in a time of uncertainty	“These were extremely time sensitive requirements because we had a workforce that was incredibly concerned, rightfully so, and anxious. We had leaders who needed answers … there was a complete vacuum of information, and quite a bit of information that we weren't sure the veracity of.” (KI 1)
3.1.2	Dissemination of evidence	“The volume of review of evidence and the utility of that [evidence] was equally amplified. I feel like in these 18 months … they've created so much. They've done so many evidence reviews and created so much awareness and understanding of that evidence. It's the equivalent of multiple years of peace time function.” (KI 1)
3.1.3	Transparent knowledge‐sharing infrastructure	“It's very useful to post all these in a transparent manner … The repository for me has been a useful way to go back to things I was looking for.” (KI 4)

#### 3.1.1 Evidence in a time of uncertainty

A major theme from the KIs was the initial paucity of COVID‐19 information and how evidence from CEST was needed in a time of uncertainty while the “evidence was still emerging.” (KI 10).

The outputs of CEST were developed in response to this need and consisted of the rapid generation of COVID‐19 reviews, comprehensive summary reports, the delivery of reports to stakeholders, and the creation of an online dashboard.

#### 3.1.2 Dissemination of evidence

The KIs emphasized the dissemination of evidence as a key deliverable from CEST and how it was used in practice. The team synthesized and condensed articles and documents from a variety of databases that were published by trusted organizations and institutions. Initially, CEST was only intended for internal use via the EOC and later became available to a wider audience within the SHA and MoH. As one participant described, “having the EOC as a vehicle to send out those requests in a written format, and the EOC receiving them, [evidence] got disseminated to a wider audience, and it did get that level of system‐learning and sharing.” (KI 9) Participants noted that as the pandemic progressed, the COVID‐19 rapid reviews were used for numerous public health strategies. CEST synthesized evidence around the impact of public health measures, best practices from other provinces, and vaccine roll‐out strategies. This included incentives for vaccine uptake and booster doses, inquiries for managing personal protective equipment (PPE), the recommended intervals between vaccine doses, breakthrough cases, sequencing, and information relating to vulnerable groups. Some participants also requested information about long COVID, the effectiveness of vaccines, and the epidemiology of variants, which became “evergreen” reviews that were regularly updated.

#### 3.1.3 Transparent knowledge‐sharing infrastructure

By building an online repository of completed COVID‐19 reviews, CEST provided a new infrastructure for knowledge to be shared across the provincial health authority. Several participants noted the utility of this resource and appreciated the convenience of returning to previous rapid review summaries. One KI described the value of a “synthesis of evidence … to present in a consumable fashion whereby decision‐makers could review it in a rapid fashion and come up with a decision.” (KI 1) Another KI emphasized how the dashboard added a layer of transparency by providing a central location for the reviews to be accessed any time.

### 3.2 Short‐term outcomes

Participants discussed several short‐term outcomes when asked about their experiences with CEST. Five themes were identified: (1) increased speed of rapid reviews, (2) increased access to reliable evidence, (3) influenced decision‐making, (4) increased confidence and reassurance, and (5) leveraged partnerships. Exemplar quotes are described in Table 2.

**TABLE 2:** Short‐term outcomes of CEST and exemplar quotes

Section	Outcomes	Exemplar quote
3.2.1	Increased speed of rapid reviews	“The Think Tank filled what was at that time, and continues to be, a mission critical role for pandemic response…. the strength of the Think Tank was the breadth of the questions that we could ask, the responsiveness, and the rapid fashion in which those responses were able to be created in order to inform our decision‐making.” (KI 1)
3.2.2	Increased access to reliable evidence	“The evidence on COVID‐19 and items around COVID‐19 was evolving so quickly that what was valid today on Friday, in two weeks, it could be completely changed… The amount of misinformation and disinformation that was available to everybody through the internet was amazing and incredibly difficult to manage, and it still is incredibly difficult to manage. The ability to actually call upon the Think Tank to review some of these items and to critically review some of the assertions made, and to debunk them, was very important.” (KI 1)
3.2.3	Influenced decision‐making	“We would, for example, look at it at our clinical expert group, and that consists of partners not just from the SHA, but also from the University, from the Ministry, et cetera. We would use this information to make appropriate recommendations. Most of the time to the government, then we hope they can accept it.” (KI 5)
3.2.4	Increased confidence and reassurance	“At the beginning of the pandemic there was a lot of value in having the Evidence Support Team because it provided reassurance to people that they were getting answers to some of these questions, and it improved the confidence in what we were writing and publishing into some of those documents. So, to me that was very important. [The staff] were afraid. The evidence or the information was coming so fast and changing so fast that it was important for people to feel that there was some expertise in what they were being told.” (KI 11)
3.2.5	Leveraged partnerships	“One of the challenges during any crisis is that subject matter experts or content experts aren't integrated into people who have to make decisions based on evidence. I think it is a good example of the College of Medicine and the department, Community Health and Epidemiology, really integrating into SHA and government information needs.” (KI 4)

#### 3.2.1 Increased speed of rapid reviews

The urgency and rapid spread of COVID‐19 necessitated faster review and synthesis of the evidence compared to traditional research timelines. In doing so, it reduced the lag time between research synthesis and adoption into practice. One informant emphasized the speed of CEST, “The timelines for these evidence reviews, in retrospect, when I think back to it, we would ask for this information, and we'd give a 24‐to‐48‐h timeline for return. That's an incredible feat when you think about it… Quite clearly, the responsiveness of the Think Tank was excellent … the volume of review of evidence and the utility of that was equally amplified” (KI 1). Another had a similar observation, “One of the strengths of the team was the rapid response and the just‐in‐time information. I think it was very valuable to have that kind of rigor.” (KI 10) Other KIs mentioned a prompt turnaround time: “I can say that the rapidity with which evidence and opinion is now going from a clinical question or a pandemic‐related or COVID‐related question and becoming published is something I've never seen in my career as a physician or clinician.” (KI 1) Another emphasized, “[the CEST reviews] were really beyond our normal standard capacity, and we were looking for better ways.” (KI 9).

#### 3.2.2 Increased access to reliable evidence

Early in the pandemic, KIs recalled the difficulty of navigating the amount of emerging information on COVID‐19. One KI described how CEST gave them access to the “right information … in the right [amount of] time.” (KI 10) Others described the efficiency of the central area for the evidence reviews, “having access to an informal way to get information that's relevant for the work that you do has been really valuable.” (KI 6). CEST was able “to synthesize the information out there and bring it to the organization in a way that can make it operational” (KI 11), providing access to a resource that was previously unavailable.

Several participants mentioned how the rapid reviews provided credible evidence and were useful for communication purposes. One informant remarked, “how I use[d] the information in practice was to inform my communication with others to link it back to evidence” (KI 3). Participants emphasized that this was useful to challenge misinformation throughout the pandemic.

#### 3.2.3 Influenced decision‐making

Overall, several informants said the CEST reviews played an important role in guiding leaders and influencing decision‐making. As one participant stated, “it did inform our decision‐making across the board.” (KI 1) CEST reviews provided evidence to the Clinical Expert Advisory group which informed the EOC and a COVID‐19 modeling team who were able to integrate the evidence with local and national data. One KI noted, “we used the information from the evidence reviews to determine the benefit on the model, and we adjusted it for Saskatchewan context.” (KI 8) A key informant (KI 4) mentioned how CEST reviews supplemented knowledge for Saskatchewan specific decisions. Another recalled how some decisions from an operational perspective in Saskatchewan had not yet been made at the national level (KI 6). Some informants also discussed how the evidence‐informed decisions by both formal and informal consultation with CEST. These methods included accessing the online repository, sharing reviews in EOC and Clinical Expert Advisory meetings, or by distributing reviews between colleagues through email. This information directly informed practice, as one KI stated:

Before we had a provincial masking mandate, there was a lot of concern about: how would we determine mask exemptions? How would we manage those? Who should be mask exempt? And what is the evidence behind this? So, I turned to the Think Tank to ask them to do a rapid review on the efficacy of masking to decrease transmission of COVID‐19. We used that information directly to create guidelines for our clinicians. (KI 1).

The KIs described how access to the CEST deliverables guided pandemic recommendations, the vaccine roll‐out campaign, guidance around PPE, vaccine hesitancy, and the implementation of public health programs. One key informant noted how the reviews guided proactive approaches to the vaccine booster rollout: “[CEST has] been really key in helping us get the international summary and international review of what people are seeing in other countries that's going to help us inform good decision‐making prior to national bodies making those decisions. We're rolling out the booster campaign now.” (KI 6) Another participant, who used the evidence reviews for the COVID‐19 vaccine implementation, commented on how it added value to their work, “[The Think Tank] always served the purpose of how we can use the data to make our strategy better.” (KI 5).

#### 3.2.4 Increased confidence and reassurance

Some KIs expressed that evidence‐based reviews from CEST gave them a sense of reassurance and confidence to make decisions. Receiving relevant and timely information was valuable at decision‐making tables and in day‐to‐day practice. As one participant described:

Having that background to know that there were evidence‐based recommendations gave me the confidence to be able to speak as a [leader], that we were following best evidence going forward and then I was able to communicate that to others. (KI 3)

#### 3.2.5 Leveraged partnerships

Several participants commented on how the formation of CEST was a collaborative effort and leveraged partnerships with colleagues. The CEST initiative brought together librarians, clinician‐scientists, and academics who contributed their unique skills to support the response. Another KI said, “The Think Tank was quite helpful in providing the long COVID evidence review, but also in bringing together the right people to discuss long COVID and to try to position the SHA in an advantageous position when considering how to deal with long COVID” (KI 1), emphasizing that the Think Tank provided a foundation for a “best evidence approach” to healthcare needs. The research and clinical expertise of the team were useful to create comprehensive reports that were commonly shared among colleagues. A participant noted how CEST was circulated “with all [their] Medical Health Officer colleagues, [which] definitely stimulated conversation.” (KI 5).

### 3.3 Long‐term outcomes

All participants agreed that there could be a future application of the CEST initiative and suggested several recommendations. These were incorporated in four prominent themes, (1) strengthening collaboration to reduce duplication, (2) upgrading system components, (3) addressing limitations within research timelines, and (4) continuing to implement CEST as an LHS (Table 3).

**TABLE 3:** Long‐term outcomes of CEST and exemplar quotes

Section	Outcome	Exemplar quote
3.3.1	Strengthening collaboration to reduce duplication	“I would suggest even the tools that are developed like quick key messages, PowerPoints…. Even having that direct connection with operations to support how that knowledge can be [translated into] actions. Otherwise, it just sits in people email. It's that gap, I think that could be a priority.” (KI 7)
3.3.2	Upgrading system components	“I think going forward we would have to figure out what is the focus of the team. Who coordinates that team? Who is the triage point? Is it linked with what level of decision‐making in the organization? So, there's lots of questions around how it would be set up and what and how it would work.” (KI 10)
3.3.3	Addressing limitations with research timelines	“…the real challenge with all research or evidence reviews is that the knowledge mobilization, the evidence to action piece.” (KI 7)
3.3.4	Implement as an LHS	“I absolutely think that there is a future state for the Think Tank within the SHA, it will inform strategy and I think it will inform efficiency, and I think it will inform best patient care that would be the function of the Think Tank in the future.” (KI 1)

#### 3.3.1 Strengthening collaboration to reduce duplication

Two participants noted that work was likely duplicated across teams and efforts between different research portfolios could have been better synchronized: “we probably need to be a bit more integrated so that we're hearing about the updates, and we're probably duplicating work.” (KI 8) Another reaffirmed this statement, “I think it was a real disconnect and a miss that our teams weren't more closely connected.” (KI 7) Strengthening the network between CEST and the data modeling team would help bolster the expertise of both.

#### 3.3.2 Upgrading system components

To improve the awareness of CEST and the online dashboard, a participant recommended creating updates or an alert to notify when new evidence is available. Another KI suggested using infographics or tools to convey quick key messages and connect with the operational side of the health system (KI 7).

#### 3.3.3 Addressing limitations with research timelines

Although CEST provided information relevant to pressing clinical and policy questions, two participants also noted that adjusting the rapid review evidence to a Saskatchewan context was challenging. Given that CEST only accessed published peer‐reviewed or grey literature, Saskatchewan‐specific data was not always contextualized with the review findings. Therefore, not all questions could be fully answered due to time constraints and the availability of evidence. One informant emphasized this outcome and how academia does not always meet desired timelines:

Research timelines don't necessarily line up with our operational timelines. As much as we like to, as a system, say that our decisions are evidence informed, often decisions are made without research, because it just takes so long to do research… the evidence reviews seem to be a bit delayed. And we also know, it's just the nature of research. (KI 7)

#### 3.3.4 Implement as an LHS

All participants agreed the program would be useful when asked about how CEST could be applied in the future, given that nothing currently exists to replace the role it can have in the health system: “I think it would be short sighted, and I think that we would not have a good foundation for new programs, or adjustment of programs that need to become better.” (KI 1) One participant (KI 10) recommended that implementing CEST sustainably into the health system would require good judgment and planning with research questions that are carefully considered. Likewise, some participants mentioned how health system leaders need to choose wisely in a system with limited resources and understand the coordination of the team to ensure it is a sustainable investment moving forward. Leveraging other skillsets within the health system where would also strengthen the capacity of the initiative. Another participant noted how health systems need “evidence‐based decisions about what we're investing in when it's one investment versus another.” (KI 3) Importantly, participants commented on how this initiative could support other health system needs beyond COVID‐19:

I could see a real close tie in terms of developing clinical standards going forward, not just related to COVID, it might not be on every clinical standard, but there is potential that a resource like the Think Tank could be reviewing upcoming questions of interest and help inform where we need to go with clinical standards, clinical appropriateness, that work, or there could be other linkages. (KI 3)

Similarly, one participant mentioned that using CEST to gather information on relevant practices and models that can be applied in Saskatchewan was more important than simply generating more research. “We don't need a whole bunch more research. What we need is to be really well informed about the best practices that are out there and what has changed since they've been relevant … there is so much best practice in healthcare that's never implemented.” (KI 6) Another KI reiterated: “I do believe that it would be valuable to have that just‐in‐time information available for clinical decision‐making and administrative decision‐making as well…. There is value moving forward and hopefully the evidence from this discussion and others will help to point that it is a valuable and useful tool.” (KI 10).

## 4 DISCUSSION

Despite a need for efficient and cost‐effective LHSs, there are only few high quality studies published about LHS implementation and evaluation.^3^, ^8^, ^30^ Our findings illustrate that the CEST initiative supported decision‐making, guided health system leaders, expedited reviews of scientific evidence, and facilitated knowledge mobilization and collaboration in the health system. The reviews supplemented the COVID‐19 modeling team data and informed provincial‐level policies, COVID‐19 vaccine implementation, and decisions around PPE, vaccine hesitancy, masking mandates, and a variety of other priority health system questions. Users described the development of the online repository as an effective and transparent way to provide access to new COVID‐19 evidence reviews. Participants also found that CEST leveraged existing relationships, skillsets, and resources to produce timely and relevant information to inform system leaders. Additionally, the CEST initiative produced rapid reviews in a matter of weeks or days, which exceeded some provincial or national programs.^31^, ^32^ This is significant considering that delays between research and practice is a well‐known challenge for health systems.^1^, ^5^, ^17^, ^33^ Participants expressed this same frustration, reinforcing that CEST was a valuable resource for them. While the rush of new publications throughout the COVID‐19 response has raised questions around the validity of some conclusions,^5^, ^17^ participants appreciated the expertise of a skilled research team for evidence synthesis. Overall, participants offered valuable feedback and saw the value of CEST to facilitate knowledge generation and inform clinical standards in Saskatchewan.

While all KIs agreed that the CEST could have future applications in the health system, several recommendations were made to help improve the initiative and achieve long‐term outcomes. To implement sustainably, factors such as limited funding and resources, coordination gaps, and optimizing applicability beyond COVID‐19 would need to be considered. This finding is important considering there has been wide support and encouragement to implement LHSs well before the COVID‐19 pandemic.^3^, ^8^, ^11^ Integrating teams was a prominent theme from the interviews and is important feedback moving forward. Similar barriers were found from an LHS evaluation in the United Kingdom,^1^ which reported key challenges around time constraints, different working cultures and priorities, and communication.^1^ Overall, the benefit of evidence‐informed decision‐making in healthcare is unanimously acknowledged in the literature,^17^, ^33^ and the COVID‐19 pandemic has been an opportunity to adapt better strategies to improve efficiency in complex systems.^6^, ^17^ Initiatives like CEST require institutional commitment, funding, and further integration within the healthcare structure.^19^ This formative evaluation assessed user feedback on the challenges and benefits of CEST to improve the design of an LHS with the intent to enhance future healthcare outcomes.

### 4.1 Strengths and limitations

Our study has several limitations, including a small sample size of KIs who were the primary users of CEST. However, given the relatively small population of Saskatchewan (1.17 million), we were able to interview informants who had the most relevant experience using CEST during the pandemic response. Because the program was implemented and evaluated in a Saskatchewan‐specific context, our results may not be generalizable to other LHSs. Given the rapidly changing landscape, not all of the KIs were aware of the prioritization process that was used by the EOC. Considering the resources available at the time, CEST would have been quickly overwhelmed had the prioritization process been expanded. Gathering support from stakeholders^34^ and conducting an evaluation,^3^ are important components for implementing a successful LHS, and therefore strength of our study was the ability to evaluate user's experiences of an LHS at a provincial level. An additional strength was studying the implementation of an LHS that comprised several organizations related to health services and planning. To the best of our knowledge, this is one of the first LHS models to be employed and evaluated in Saskatchewan.

## 5 CONCLUSION

This formative evaluation explored user experiences with a COVID‐19 knowledge‐to‐practice initiative to assess its outputs and outcomes and describe how it might serve as a functional LHS in Saskatchewan. By conducting KI interviews, we found that implementing an LHS was valued as a sustainable initiative at a provincial level in Saskatchewan. Through the evaluation of an LHS initiative, users and collaborators can understand whether a program has the desired outcomes or not. Therefore, this formative evaluation provided an opportunity to improve the implementation of an LHS initiative.

## FUNDING INFORMATION

This study was funded by the College of Medicine Research Award (CoMRAD), University of Saskatchewan. Grant/Award number: 353905.

## CONFLICT OF INTEREST

The authors declare that there is no conflict of interest.
